# Comparison between Zip-Type Skin Closure Device and Staple for Total Knee Arthroplasty: A Meta-Analysis

**DOI:** 10.1155/2021/6670064

**Published:** 2021-05-13

**Authors:** Peng Tian, Yao-min Li, Zhi-jun Li, Gui-jun Xu, Xin-long Ma

**Affiliations:** ^1^Department of Orthopedics, Tianjin Hospital, No. 406, Jiefang Nan Road, Tianjin, China 300211; ^2^Department of Rehabilitation, Tianjin Hospital, No. 406, Jiefang Nan Road, Tianjin, China 300211; ^3^Department of Orthopedics, Tianjin Medical University General Hospital, No. 154, Anshan Road, Tianjin, China 300052

## Abstract

**Purpose:**

To compare the efficacy and safety of zip-type skin closure device (SCD) and staple in total knee arthroplasty (TKA).

**Methods:**

Potential academic articles were identified from PubMed, Springer, ScienceDirect, and Cochrane Library from the inception of electronic databases to July 2020. The statistical analyses were performed with RevMan 5.1.

**Results:**

One randomized controlled trial (RCT) and 5 non-RCTs met the inclusion criteria. Present meta-analysis reveals that SCD is associated with lower wound pain score, scar score, and readmission compared with a staple. No significant differences are identified in terms of wound total complications, dehiscence, blisters, and infection.

**Conclusions:**

Comparing with a staple, zip-type SCD is a less painful skin closure method with fewer medical cost undergoing TKA.

## 1. Introduction

Total knee arthroplasty (TKA) has become a well-accepted surgical treatment for end-age arthritic knee problems. Wound closure is an increasing research focus in TKA [1]. Secure wound closure is crucial to minimize wound complications and facilitate immediate rehabilitation [2]. Surgical staple has been adopted for surgical skin closures for decades [3]. A conventional surgical staple during TKA has been well established in the literature [4]. In 2017, Kim and his colleague [5] performed a meta-analysis show that skin closure with staples provides lower incision complications, less incision closure times, and an overall reduction in resource utilization. However, negative effects were still reported, such as feeling pain and fear when staples were removed [6, 7]. Tseng et al. [8] even applied topical anesthesia for staple removal after TKA to relieve pain. Additionally, Singh et al. [7] suggest that staples are associated with bleeding, infections, and additional scar formation after staple removal.

A zip-type skin closure device (SCD), atraumatic and noninvasive, has been developed utilizing adhesive strips on either side of the wound, with zip ties traversing the wound that are linked in a zigzag pattern between adjacent rows [9]. Several studies [10–15] have compared SCD with staples in patients undergoing TKA. However, whether SCD is safe and efficient in TKA remains controversial. Therefore, the purpose of this study is to systematically review the current evidence in the literature to compare the efficacy and safety of SCD with a staple in patients undergoing TKA.

## 2. Methods

### 2.1. Search Strategy

Potentially relevant published academic literatures were searched out from PubMed, Springer, ScienceDirect, and Cochrane Library from the inception of electronic databases to July 2020. The secondary sources were identified from the included literatures in the references. None of the studies was excluded by language. The searching keywords used were “replacement OR arthroplasty”, “knee”, “skin closure tape”, and “staple”.

### 2.2. Selection Criteria and Quality Assessment

The present meta-analysis was enrolled in published randomized controlled trials (RCTs) and non-RCTs comparing SCD with staples in the patients undergoing primary TKA. Two independent reviewers determined the suitability of the literatures. And the third reviewer resolved the disagreements. The methodological quality of RCTs was evaluated using the modification of the generic evaluation tool described in the Cochrane Handbook for Systematic Review of Interventions [16]. The methodological quality of non-RCTs was assessed by Methodological Index for Nonrandomized Studies (MINORS) [17].

### 2.3. Data Extraction

The data were extracted from the included literature by two independent reviewers. The incomplete data was consulted for details by writing to the corresponding author of included literatures. The following information: first author's name, publication year, intervening measures, comparable baselines, and outcome measures were extracted. Other relevant parameters were also extracted from individual studies.

### 2.4. Data Analysis and Statistical Methods

RevMan 5.1 (The Cochrane Collaboration, Oxford, United Kingdom) was used for analyzing the pooled data. The values of *P* were used to estimate the heterogeneity and *I*^2^ depending on the standard chi-square test. When *I*^2^ > 50%, *P* < 0.1 is considered to indicate significant heterogeneity, which used a random-effects model for the data analyzing. When *I*^2^ < 50%, *P* > 0.1 is considered to indicate no significant heterogeneity. A fixed-effects model was used for the data analyzing, in which no significant heterogeneity was found. Subgroup analysis was performed when the significant heterogeneity was found to investigate the sources. The mean differences (MDs) and 95% confidence intervals (CIs) were determined for continuing outcomes. The dichotomous data were calculated by the risk differences (RDs) and 95% CIs.

## 3. Results

### 3.1. Search Results

**Figure 1:**
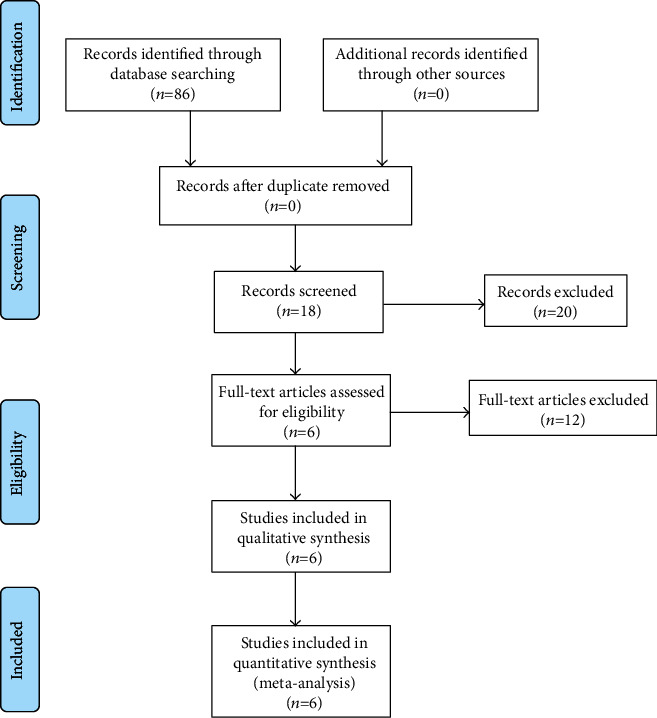
Flowchart of the study selection process.

A total of 86 studies were identified as potentially relevant literature reports. After a thorough screening of title and abstract, 80 reports were excluded according to the eligibility criteria. No additional studies were obtained after the reference review. Finally, one RCT and 5 non-RCTs [10–15] met the inclusion criteria for data extraction and meta-analysis. The search process is shown in Figure 1.

### 3.2. Risk of Bias Assessment

**Figure 2:**
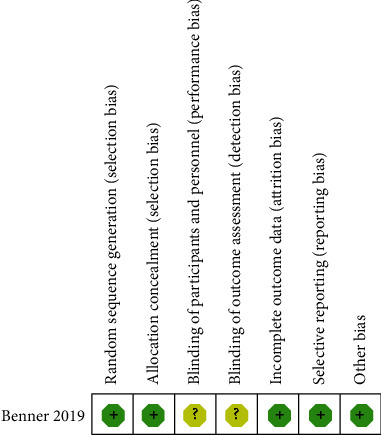
Risk of bias summary of randomized controlled trials.

The methodological quality of RCT was assessed by *Cochrane Handbook for Systematic Review of Interventions* (Figure 2). The RCT stated clear inclusion and exclusion criteria. Included RCT reported adequate methodology of randomization, concealment of allocation, and intent-to-treatment analysis. In addition, blinding was not described in the included RCT. No unclear bias was reported due to incomplete outcome data or selective outcomes. MINORS scores of non-RCTs range from 20 to 22. The methodological quality assessment of non-RCTs is presented in Table 1.

### 3.3. Study Characteristics

Demographic characteristics and other details of the included studies are presented in Table 2. In each study, the baseline characteristics of the two groups were similar.

### 3.4. Outcomes of Meta-Analysis

It was possible to perform a meta-analysis with 7 outcomes (Table 3). SCD is associated with less wound pain evaluation (MD = −1.68, *P* = 0.0001), wound scar score (MD = −1.80, *P* = 0.002), and readmission (RD = −0.02, *P* = 0.02) compared with a staple. There were no statistically significant differences between SCD and staple group for wound total complication (RD = 0.02, *P* = 0.76), wound dehiscence (RD = 0.00, *P* = 1.00), wound blisters (RD = 0.10, *P* = 0.42), and wound infection (RD = −0.00, *P* = 0.18).

## 4. Discussion

Our meta-analysis incorporated six studies and 3550 knees during primary TKA. The purpose of our meta-analysis was to assess zip-type SCD compared with surgical staples for wound closure undergoing TKA. In this analysis of studies, we found that zip-type SCD compared with surgical staples decrease wound pain evaluation, wound scar score, and readmission. Our meta-analysis was more systematic, comprehensive, and novel than the previous meta-analysis [18].

Complete wound healing without complication is an important factor for determining patient satisfaction and overall outcome in TKA [13]. Moreover, skin closure in TKA is higher tissue tension and motion. Our meta-analysis showed wound total complications, dehiscence, blisters, and infection in the zip-type SCD group are not higher than those in the staple group. These results are consistent with previous studies. In theory, zip-type SCD creates a zone of isolation at the wound edge by transferring the stress of knee flexion away from the skin incision. Furthermore, the zigzag interlocking linkage produces gentle compression at the wound edge [9]. We concluded that zip-type SCD may be an acceptable alternative to staple during TKA.

It is reported that patients may feel pain and fear during staple removal and could suffer from bleeding, additional dressing, superficial infections, or scar formation on piercing sites after staple removal [6, 7]. The zip-type SCD involves no needles and sharps and does not puncture the skin [13]. Present meta-analysis showed that zip-type SCD provide less pain and scar score comparing with a staple. The zip-type SCD is more comfortable for the patients, with no pain upon removal and no suture marks after removal.

Previous studies indicated that the application of zip-type SCD decrease mean direct hospital total costs after primary TKA. Tayamaka et al. [15] and Carli et al. [12] reported that cost for zip-type SCD is lower than a staple. Recently, Alnachoukati et al. [10] reported that wound closure with zip-type SCD in TKA significantly decreases the amount of wound-related phone calls, wound-related emergency room admits, and antibiotics prescribed due to wound complications than the staple group. They found that staples require more cost calculated per patient compared with the zip-type SCD group ($228 versus $50, respectively). Furthermore, Sutton et al. [14] showed that the use of zip-type SCD decreases the length of hospital stay, resource-intensive discharge status, and rates of all-cause readmission as compared with skin staples. Pooled results indicated that the readmission rate was lower in zip-type SCD groups. Zip-type SCD is more economical than staple with similar efficacy. Therefore, there is an advantage for patients undergoing TKA to use zip-type SCD.

Several potential limitations should be noted. (1) Only one RCT and 5 non-RCTs were included, and the sample size of all studies was relatively small; (2) methodological quality of included studies and insufficient outcomes may weaken our analysis; (3) we failed to perform subgroup analysis and determine the source of heterogeneity for the limited number of included studies.

## 5. Conclusion

Comparing with a staple, zip-type SCD is a less painful skin closure method with fewer medical cost undergoing TKA. Therefore, more high-quality research is required to determine the effectiveness and safety of SCD in TKA.

## Figures and Tables

**Table 1:** Quality assessment for nonrandomized trials.

Quality assessment for nonrandomized trials	Alnachoukati 2019	Carli 2017	Ko 2017	Sutton 2018	Takayama 2017
A clearly stated aim	2	2	2	2	2
Inclusion of consecutive patients	2	2	2	2	2
Prospective data collection	0	2	0	2	2
Endpoints appropriate to the aim of the study	2	2	2	2	2
Unbiased assessment of the study endpoint	2	2	2	2	2
A follow-up period appropriate to the aims of study	2	2	2	2	2
Less than 5% loss to follow-up	2	2	2	2	2
Prospective calculation of the sample size	0	0	0	0	0
An adequate control group	2	2	2	2	2
Contemporary groups	2	2	2	2	2
Baseline equivalence of groups	2	2	2	2	2
Adequate statistical analyses	2	2	2	2	2
Total score	20	22	20	22	22

**Table 2:** Characteristics of included studies.

Study	Design	Intervention	Cases	Mean age	Female	BMI	Follow-up
Alnachoukati 2019	RCS	SKD Staple	65 65	66.8 68.3	42 40	34.3 31.4	1 month
Benner 2019	RCT	SKD Staple	25 25	66.4 66.4	12 12	NS	2 months
Carli 2017	PCT	SKD Staple	221 1001	60.8 65.3	121 639	31.4 29.6	6 weeks
Ko 2017	RCS	SKD Staple	45 45	68.8 70.38	38 34	24.9 24.42	3 months
Sutton 2018	RCS	SKD Staple	971 971	65.1 65.5	621 612	NS	3 months
Takayama 2017	RCS	SKD Staple	38 38	73.9 73.8	33 31	25.9 26.5	3 months

RCS: retrospective controlled trial; RCT: randomized controlled trial; PCT: prospective controlled trial; SKD: skin closure device; F: female; NS: not state.

**Table 3:** Meta-analysis results.

Outcome	Studies	Groups (SCD/staple)	Overall effect			Heterogeneity	
			Effect estimate	95% CI	*P* value	*I* ^2^ (%)	*P* value
Total wound complications	4	369/1149	0.02	-0.10, 0.14	0.76	82	0.0009
Wound dehiscence	4	173/173	0.00	-0.03, 0.03	1.00	0	0.86
Wound blisters	2	259/1039	0.10	-0.14, 0.35	0.42	86	0.008
Wound infection	3	1081/1081	-0.00	-0.01, 0.00	0.18	0	0.39
Wound pain evaluation	2	70/70	-1.68	-2.08, -1.28	0.0001	95	0.0001
Wound scar score	2	70/70	-1.80	-2.96, -0.64	0.002	77	0.04
Readmission	2	55/1972	-0.02	-0.04, -0.00	0.02	0	0.80

SCD: skin closure device; CI: confidence interval.

## Data Availability

The data generated during the current study are available from the corresponding author upon reasonable request.

## Supplementary Materials

Supplementary Materials Supplement 1: forest plot. Figure S1: forest plot diagram showing total wound complications. Figure S2: forest plot diagram showing wound dehiscence. Figure S3: forest plot diagram showing wound blisters. Figure S4: forest plot diagram showing wound infection. Figure S5: forest plot diagram showing wound pain evaluation. Figure S6: forest plot diagram showing wound scar score. Figure S7: forest plot diagram showing readmission. Supplement 2: funnel plot. Figure S1: funnel plot diagram showing total wound complications. Figure S2: funnel plot diagram showing wound dehiscence. Figure S3: funnel plot diagram showing wound blisters. Figure S4: funnel plot diagram showing wound infection. Figure S5: funnel plot diagram showing wound pain evaluation. Figure S6: funnel plot diagram showing wound scar score. Figure S7: funnel plot diagram showing readmission.
