# Supplementary material for: Comparison between Zip-Type Skin Closure Device and Staple for Total Knee Arthroplasty: A Meta-Analysis
Source: Biomed Res Int. 2021 May 13;2021:6670064. doi: 10.1155/2021/6670064 (PMC8137286; doi:10.1155/2021/6670064)
Supplement: Supplementary Materials — Supplement 1: forest plot. Figure S1: forest plot diagram showing total wound complications. Figure S2: forest plot diagram showing wound dehiscence. Figure S3: forest plot diagram showing wound blisters. Figure S4: forest plot diagram showing wound infection. Figure S5: forest plot diagram showing wound pain evaluation. Figure S6: forest plot diagram showing wound scar score. Figure S7: forest plot diagram showing readmission. Supplement 2: funnel plot. Figure S1: funnel plot diagram showing total wound complications. Figure S2: funnel plot diagram showing wound dehiscence. Figure S3: funnel plot diagram showing wound blisters. Figure S4: funnel plot diagram showing wound infection. Figure S5: funnel plot diagram showing wound pain evaluation. Figure S6: funnel plot diagram showing wound scar score. Figure S7: funnel plot diagram showing readmission. [file 6670064.f1.docx]

**Supplement 1**


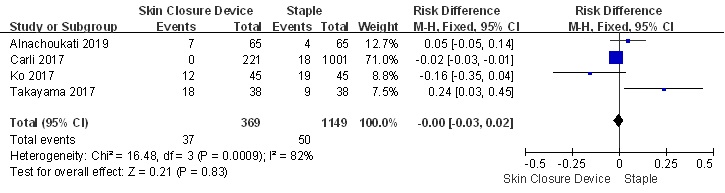


Figure S1 Forest plot diagram showing total wound complications


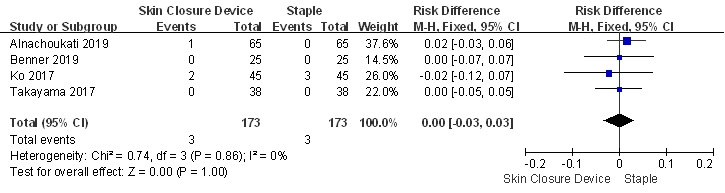


Figure S2 Forest plot diagram showing wound dehiscence


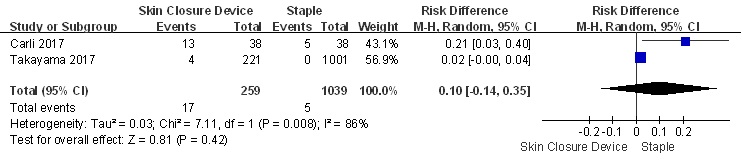


Figure S3 Forest plot diagram showing wound blisters


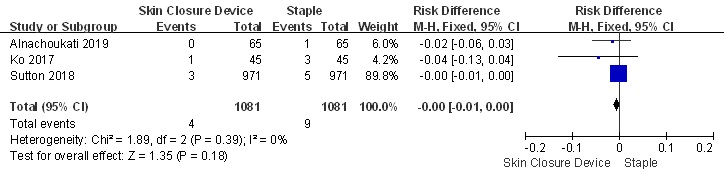


Figure S4 Forest plot diagram showing wound infection


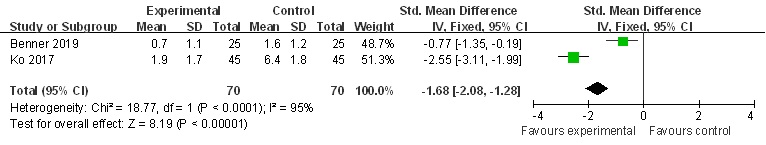


Figure S5 Forest plot diagram showing wound pain evaluation


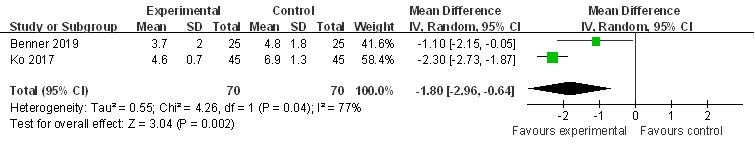


Figure S6 Forest plot diagram showing wound scar score


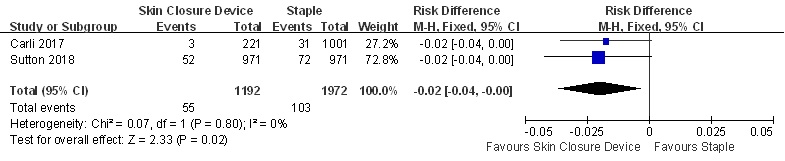


Figure S7 Forest plot diagram showing re-admission

**Supplement 2**


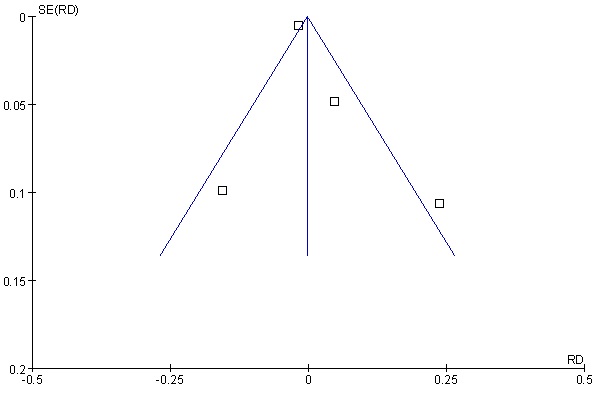


Figure S1 Funnel plot diagram showing total wound complications


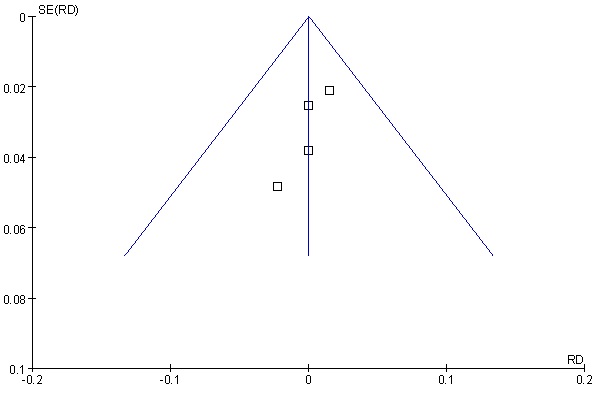


Figure S2 Funnel plot diagram showing wound dehiscence


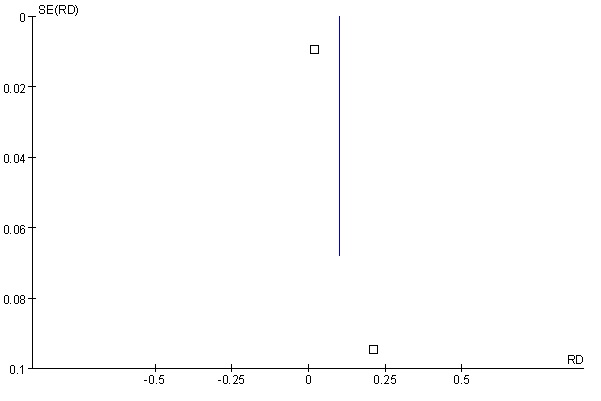


Figure S3 Funnel plot diagram showing wound blisters


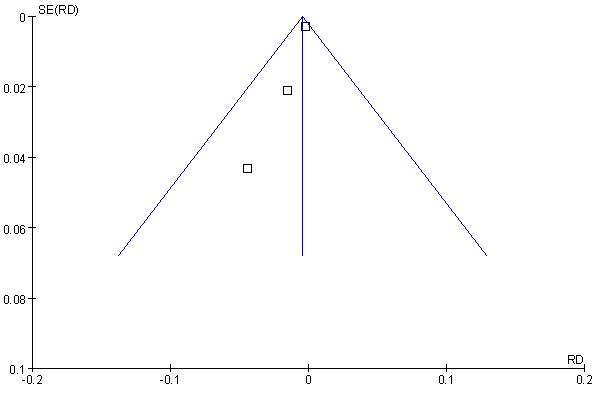


Figure S4 Funnel plot diagram showing wound infection


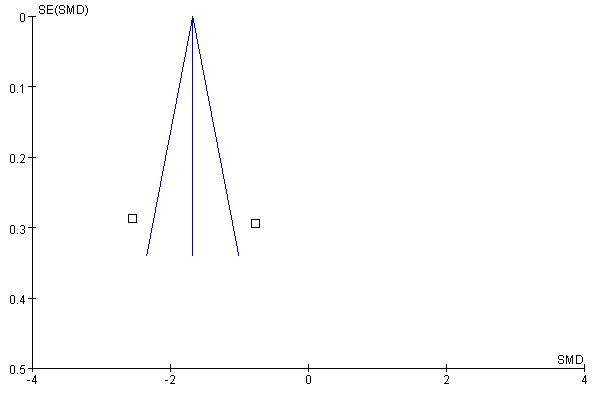


Figure S5 Funnel plot diagram showing wound pain evaluation


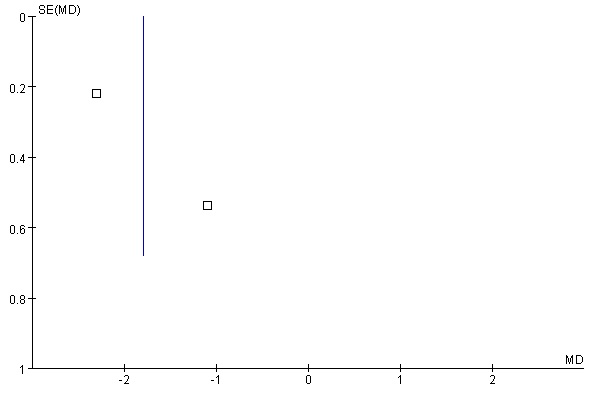


Figure S6 Funnel plot diagram showing wound scar score


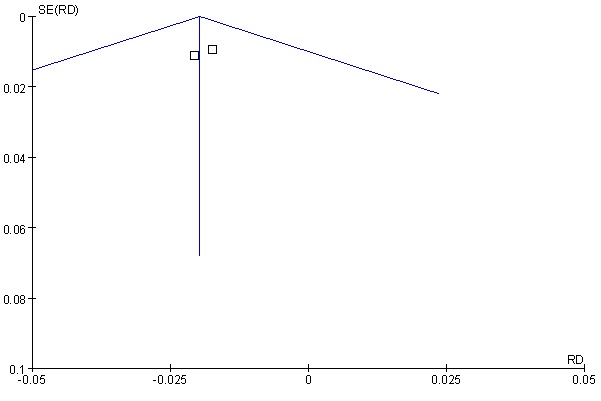


Figure S7 Funnel plot diagram showing re-admission
